# Peer learning in the UNSW Medicine program

**DOI:** 10.1186/s12909-015-0450-y

**Published:** 2015-10-02

**Authors:** Helen A. Scicluna, Anthony J. O’Sullivan, Patrick Boyle, Philip D. Jones, H. Patrick McNeil

**Affiliations:** 1 UNSW Medicine, UNSW Australia, Sydney, Australia; 2 Q Associates, UNSW Australia, Sydney, Australia; 3 Faculty of Medicine and Health Sciences, Macquarie University, Sydney, Australia

**Keywords:** Peer learning, Near-peer teaching, Teamwork, Leadership, Deep learning

## Abstract

**Background:**

The UNSW Australia Medicine program explicitly structures peer learning in program wide mixing of students where students from two adjoining cohorts complete the same course together, including all learning activities and assessment. The purpose of this evaluation is to explore the student experience of peer learning and determine benefits and concerns for junior and senior students.

**Methods:**

All medical students at UNSW Australia in 2012 (*n* = 1608) were invited to complete the Peer Learning Questionnaire consisting of 26 fixed-response items and 2 open-ended items exploring vertical integration and near-peer teaching. Assessment data from vertically integrated and non-vertically integrated courses were compared for the period 2011–2013.

**Results:**

We received valid responses from 20 % of medical students (*n* = 328). Eighty percent of respondents were positive about their experience of vertical integration. Year 1 students reported that second year students provided guidance and reassurance (87.8 %), whilst year 2 students reported that the senior role helped them to improve their own understanding, communication and confidence (84 %). Vertical integration had little effect on examination performance and failure rates.

**Conclusions:**

This evaluation demonstrates that vertical integration of students who are one year apart and completing the same course leads to positive outcomes for the student experience of learning. Students benefit through deeper learning and the development of leadership qualities within teams. These results are relevant not only for medical education, but also for other professional higher education programs.

## Background

More than a decade ago, UNSW Australia introduced an innovative educational program to graduate medical doctors with the capabilities needed for 21st century professional practice [1]. The 6-year undergraduate entry, fully integrated, outcomes-based UNSW Medicine program contains many innovative features, including a process aimed at selecting students with the appropriate attributes and motivation to study medicine [2], the development of generic capabilities as a core for the learning and practice of medical expertise [3, 4], a graded learning process to progressively develop student autonomy, an in-depth research experience for all students [5], a comprehensive assessment system designed to ensure strong alignment between teaching, learning and outcomes [6–8], and an emphasis on experiential learning and a collaborative learning environment. All these measures were designed to prepare graduating doctors for the demands of the ever evolving complex health system.

In this report we describe an evaluation of one of the most innovative aspects of the UNSW Medicine program–explicitly structured peer learning, a term that describes students working together to acquire knowledge, skills and attitudes by helping each other to learn, and learning by helping each other [9]. Vertical integration of learners (learning groups of students who are one year apart in their medical education), and near-peer teaching (teaching that occurs between students who are in the same program but who are one to six years apart) are two forms of peer learning that have been evaluated. The adoption of vertical integration to teach students was also designed to expose students to teamwork with senior and junior colleagues, a situation they will face as junior doctors.

The UNSW Medicine program contains no discipline-specific biomedical courses, but is composed of 4 to 8-week blocks of fully integrated courses or terms based around broad themes. For example, during the first two years, two of the integrated 8-week courses are ‘Beginnings, Growth and Development’ (BGD) and ‘Health Maintenance’ (HM). Themes for student learning in these courses include ‘Childhood growth and development’ and ‘Nutrition, growth and body image’ in BGD, and ‘Homeostasis, sustenance and equilibrium’ and ‘Lifestyle factors that risk health’ in HM [1].

One of the most unique and distinctive features of the UNSW Medicine program is bringing together students from adjoining years for all learning and assessment activities in the same course [1]. For example, students in Years 1 and 2 complete semester 1 courses (including BGD) within their cohort. However the two cohorts are combined in the semester 2 courses (including HM), that is Year 1 and 2 students complete the same learning activities and assessments as one combined cohort. Moreover, students from the ‘senior’ year are encouraged to take on leadership roles within student groups. In implementing this structure we also sought to develop students’ teamwork skills that they would require when working in healthcare teams where clinicians with differing levels of expertise and experience work together.

From the original design phase, we outlined four key aims of creating a curriculum structure that incorporated the explicit vertical integration of learners [1]. Three of these concerned the development of teamwork, one of the key generic capabilities that represent the UNSW Medicine core. We hypothesised that students learning together in small group (e.g. tutorial), and medium group (e.g. laboratory class) settings would encourage collaborative learning, create structures for peer support, mentoring and modelling of behaviour and learning approaches, and importantly provide some leadership experience for students that mirrored real healthcare teams where different levels of post-graduation doctors work and learn together. This latter aim is particularly important for recently graduating medical practitioners, where there exists a strong mentoring dynamic in the working medical team between junior medical officers/house officers, hospital registrars/fellows, and consultant/attending doctors, a concept referred to by Shulman [10], as ‘professional signature pedagogies’. While much effort has been invested to develop cross-professional or inter-disciplinary teamwork between health professionals [11–13], there is relatively less literature on approaches at university that provide students with experiences of being both junior and senior members within a single discipline that would assist them negotiate the extant dynamic of being both a junior and a senior member in real world medical teams [14].

The final aim of learner vertical integration articulated in 2006 was to enhance the richness of knowledge structures created by learners, by providing opportunities for reiteration and refinement of knowledge in the light of new experience [1]. Designing educational programs that encourage students to adopt deep approaches to learning, aiming for higher order understanding, and application of learning in new contexts has been shown to be very difficult [15, 16]. Nevertheless, there is evidence that near-peer teaching involving tutoring more junior students is one way to encourage deeper learning by the more senior students [17–19], as well as improving learning for the juniors [20].

With these considerations in mind, our evaluation of peer learning at UNSW Australia set out to explore the student experience of structured vertical integration of learners, and determine benefits and concerns for junior and senior students.

## Methods

### The peer learning questionnaire

A questionnaire to evaluate peer learning was developed following a comprehensive literature review and focus groups with medicine students in years 4–6. The focus groups were transcribed and discussed by the research team to identify the terminology used and understood by students in relation to peer learning and to explore students’ perceptions of vertical integration and near-peer teaching. Questionnaire items were generated and critiqued by the research team, medical education specialists and student representatives in an iterative process until agreement was reached on the 26 fixed-response items (4 point Likert scale; strongly agree-strongly disagree) and 2 open-ended items forming the Peer Learning Questionnaire.

The Peer Learning Questionnaire has a response-driven pathway where students’ progression through the questionnaire varies based on their year in the program and experiences of peer learning. Items 1–12 and 2 open-ended items explore students’ reflections on their experience of peer learning in years 1 and 2 only. Items 13–19 explore senior students’ experience of peer learning in years 1–6 and items 20–26 explore students’ experience of voluntary near-pear teaching in years 1–6. Individual items from the Peer Learning Questionnaire are shown in Tables 2, 3 and 6. All students answered the question ‘All things considered how do you rate your level of satisfaction with your experience as a student in medicine at UNSW?’

### Participants

The participants in this study were the 2012 UNSW Australia medical students in years 1–6 who received an email inviting them to complete the Peer Learning Questionnaire online. All students answered items 1–12 and 2 open-ended items, students in years 2–6 answered items 13–19, students in years 3–6 with near-peer teaching experience answered items 20–25 and students without near-peer teaching answered items 20 and 26. Students in years 1–5 completed the questionnaire in October 2012, prior to the completion of their final year examinations, whereas students in year 6 completed the questionnaire in November 2012 after they had completed their final medical school examinations.

### Assessment data

Assessment data for the vertically-integrated HM Course and the non-vertically integrated BGD Course were compared for the period 2011–2013.

### Analysis

The qualitative data from the open-ended items ‘The best features of vertical integration are’ and ‘The features of vertical integration that need improving are’ were analysed using an inductive design based on thematic analysis without any a priori researcher theoretical bias. After multiple readings and interpretations of the open-ended responses, themes were identified from the data which were discussed by the researchers in an iterative process and manually coded using NVivo. Coding of the data continued until all themes were identified and discrepancies in coding were resolved by discussion between the researchers [21].

The ‘strongly agree’ and ‘agree’ responses for each fixed-response item were combined and converted to a percentage of the total response and reported as ‘agreement’. Mean end of course examination grades were calculated for the HM and BGD Courses for the period 2011–2013. Statisical analysis (logistic regression) was completed using SPSS (version 22).

### Ethics

Ethical approval for this study was obtained from the UNSW Medical and Community Human Research Ethics Advisory Panel (Approval no: 2012-7-46).

## Results

### Students find peer learning a positive experience

Valid questionnaire responses were received from a total of 328 students, representing 20 % of the total year 1–6 student cohort. All years were well represented in respondents with approximately 50 students responding from each of years 1–6. The response-driven pathway results in students from different years answering different numbers of questions (Table 1). A total of 379 responses were received on the 2 open-ended items; 207 comments on the best features of vertical integration and 172 comments on the features of vertical integration that need improving. Females comprised 58.2 % of respondents, similar to the gender balance of the entire cohort (53.1 % female).

**Table 1:** Peer learning questionnaire response-driven pathway and response rate

Peer learning questionnaire items	Participants	No. of respondents by total cohort and year % (n)		No. in cohort
Items 1–12 2 open-ended items	years 1–6	years 1–6	20.4 % (*n* = 328)	1608
		year 1	19 % (*n* = 51)	269
		year 2	20.4 % (*n* = 55)	269
		year 3	20.9 % (*n* = 53)	253
		year 4	21.1 % (*n* = 60)	284
		year 5	19.9 % (*n* = 56)	282
		year 6	21.1 % (*n* = 53)	251
Items 13–19	years 2–6	years 2–6	20.7 % (*n* = 277)	1339
Item 20	years 3–6	years 3–6	20.7 % (*n* = 222)	1070
				No. of respondents (years 3–6)
Items 21–25	years 3–6 with near peer teaching experience	years 3–6	54.1 % (*n* = 120)	222
		year 3	39.6 % (*n* = 21)	53
		year 4	50 % (*n* = 30)	60
		year 5	57.1 % (*n* = 32)	56
		year 6	69.8 % (*n* = 37)	53
Item 26	years 3–6 without near peer teaching experience	years 3–6	45.9 % (*n* = 102)	222
		year 3	60.4 % (*n* = 32)	53
		year 4	50 % (*n* = 30)	60
		year 5	42.9 % (*n* = 24)	56
		year 6	30.2 % (*n* = 16)	53

More than 80 % of respondents expressed positive satisfaction with their overall experience to date of the Medicine program, which was equivalent to the 78 % of 2695 students who reported positive overall satisfaction with the Medicine program in broader student experience surveys conducted every 2 years between 2006 and 2012 [22]. Thus, we concluded that respondents to the peer learning questionnaire were generally representative of the larger UNSW Medicine student cohort.

Eighty percent of respondents were positive [either satisfied (57 %) or highly satisfied (23 %)] about their experience of structured vertical integration. When analysing responses from particular year cohorts, year 3 students were the most positive (90 %) and year 4 students the least positive (70 %) about vertical integration. Taken overall, the results indicate that students regard explicit vertical integration of learners from differing year cohorts as being an inherent and positive part of their experience of the UNSW Medicine program.

### Vertical integration of students has beneficial effects

Students’ perceptions of the structured and explicit mixing of students from adjoining cohorts were positive across all years. As shown in Table 2, more than three quarters of all students reported that year 2 students provided guidance and reassurance, helped with understanding, and provided good explanations. Interestingly, a majority (56.7 %) of students felt the presence of year 2 students motivated them to achieve higher learning goals, even though they also recognised sometimes feeling left behind by their year 2 peers (58.5 %). Nevertheless, year 1 students were not overly reliant on the presence of year 2 students for their learning, with less than half (47.9 %) agreeing that year 2 students provided guidance on reading efficiently and almost a quarter of students (22.6 %) experiencing their year 2 counterparts to be disinterested in helping them learn (Table 2).

**Table 2:** Experience of first year students

Second year students…	Agreement (%)	Mean score^a^ (SD)
Provided guidance and reassurance about how to complete the group project.	87.8	1.8 (0.7)
Helped me understand and negotiate the capability system.	75.9	2.1 (0.7)
Helped me by providing good explanations.	78.1	2.1 (0.7)
Generally weren’t interested in helping first year students.	22.6	2.9 (0.7)
Knew more and I sometimes felt left behind.	58.5	2.3 (0.8)
Motivated me to reach a higher standard than otherwise would have been the case.	56.7	2.4 (0.9)
Provided valuable guidance and advice on what to read and how to read it efficiently.	47.9	2.5 (0.8)

^a^1 = strongly agree, 4 = strongly disagree

The experience of year 2 students in mixed peer learning groups was also overwhelmingly positive. As shown in Table 3, more than 84 % of students in years 2–6 reported the senior student role was not only enjoyable but also helpful in improving their own understanding, and there were significant perceived benefits with communication and confidence. Some students (20.2 %) expressed concern that explicit near cohort mixing was an impediment to their learning as it slowed down their learning in order to teach the first year students. Overall though, more than 80 % of responses from all students agreed that vertical integration had a positive effect on their learning (Table 3).

**Table 3:** Experience of second year students

As a second year student…	Agreement (%)	Mean score^a^ (SD)
I enjoyed teaching the first year students.	89.2	1.8 (0.6)
Teaching first year students helped me clarify and deepen my knowledge and understanding of a topic.	84.8	1.9 (0.7)
Teaching first year students improved my confidence to help others with their learning.	81.6	1.9 (0.7)
Teaching first year students improved my ability to communicate effectively.	77.3	2.0 (0.7)
I felt motivated to help students in the years below me.	96.3	1.7 (0.6)
I found my learning slowed down in order to teach the first year students.	20.2	2.9 (0.7)

^a^1 = strongly agree, 4 = strongly disagree

An analysis of assessment data suggests that near-cohort mixed learning had little effect on examination performance of particular year cohorts. In the vertically-integrated HM Course, year 2 students on average, performed better than year 1 students with a mean end-of-course MCQ examination grade over the 2011–2013 period of 69.9 versus 64.0 % for year 1 students (Table 4). This compares to the non-vertically-integrated BGD Course where the mean grades for each cohort were 65.2 and 66.8 % for year 2 and year 1 students respectively. In this 3-year period, 2.8 % of students failed the HM course, of which three quarters were year 1 students. However, these failure rates in HM were not different to those for year 1 and 2 students in the non-vertically-integrated BGD courses where the combined failure rate was 3.1 % similar to 2.8 % in HM (Table 4). Near-cohort mixed learning (vertically integrated or non-vertically integrated) was not a predictor of examination performance in logistic regression analysis. Students’ year was the best predictor of performance, with year 1 students more likely to fail regardless of the structure of the course (Table 5).

**Table 4:** Mean assessment results and failure rates of students studying the phase 1 Beginnings Growth and Development (BGD) and Health Maintenance (HM) courses 2011–2013

	Year 1	Year 2	Combined/average
Beginnings Growth and Development Course (BGD)			
Mean grades	66.8	65.2	
Failure rates	5.1 %	0.9 %	3.1 %
Health Maintenance Course (HM)			
Mean grades	64.0	69.6	66.8
Failure rates	4.3 %	1.4 %	2.8 %

**Table 5:** Odds ratio, confidence intervals, beta values (standard errors) for predictor variables in logistic regression

		95 % CI for Odds Ratio		
Included	B (SE)	Lower	Odds Ratio	Upper
Constant	3.03 (0.16)			
Year	1.46 (0.27)*	2.58	4.31	7.24
Course	−0.059 (0.21)	0.62	0.94	1.42

*p < 0.01

### Near peer learning develops leadership skills within a teamwork context

The beneficial effects on learning for year 2 students within near-cohort mixed learning groups have led to the development of a clear leadership role. As seen in Table 6, 80.1 % of respondents reported that the presence of year 1 students within the same tutorial and study groups encouraged them to take on a leadership role. Furthermore, the instructional design of having near-cohort mixed learning groups has led to a dramatic expansion of student-initiated informal near-peer teaching, an activity that 68.3 % of respondents indicated was a direct effect of structured vertical integration in the Program. More than half (54.1 %) of respondents had engaged in near-peer teaching, and between 86.7 and 94.2 % of these indicated major personal benefits for their own learning (Table 6). Examples of near-peer teaching that UNSW Medicine students have spontaneously organised and engaged in range from examination preparation and revision, study groups, bed-side clinical tutorials, individual tutoring and general advice. Two thirds of respondents who had not engaged in near-peer teaching cited a lack of confidence in their own knowledge and skills as the primary reason for why they had not undertaken this activity.

**Table 6:** Development of leadership skills

Questionnaire item	Agreement (%)	Mean score^a^ (SD)
Having first year students in the scenario group encouraged me to take on a leadership role as a second year.	80.1	2.0 (0.8)
Vertical integration encouraged me to organise near-peer teaching.	68.3	2.2 (0.8)
Being a near-peer teacher motivated me to learn at a deeper level.	86.7	1.9 (0.6)
Being a near-peer teacher clarified and/or enhanced my own knowledge and skills.	94.2	1.7 (0.6)
Being a near-peer teacher improved my confidence helping others to learn.	92.5	1.8 (0.6)
I believe that students benefitted from my teaching.	99.2	1.8 (0.4)

^a^1 = strongly agree, 4 = strongly disagree

### Thematic analysis of open-ended comments

The key positive themes identified from the open-ended comments were peer support, near-peer teaching promoting deeper learning, leadership and teamwork. The negative themes were students’ resistance to peer learning, transmission of inaccurate information and impediments to learning. The following quotes are illustrative of the key themes.

#### Peer support

Many students recalled the importance and acceptance of supportive peers who were willing to provide guidance and feedback, particularly when settling into the Medicine program, as indicated by the following comments.

*As a first year I love the guidance from the second years. Especially in SGS (scenario group sessions) and hospital (tutorials). I think vertical integration is unequivocally positive. Having someone with more experience makes challenges…easier and I feel more capable of meeting those challenges. [Year 1 student]*

*The second years raise the standard that is expected, both in group projects and generally in terms of studying the courses. In my experience, the second years have always been extremely helpful and have gone out of their way to teach me and other first years. [Year 1 student]*

#### Near-peer teaching promoting deeper learning

Year 2 students enjoyed helping year 1 students through informal teaching and by passing on advice about many aspects of the Medicine program, but most importantly advice on assignments and examinations. They also reported their own knowledge was deepened through the process of teaching year 1 students.

*I enjoyed teaching the first years myself when I was a second year, as it allowed me to clarify my knowledge. It is much better to learn by teaching, as you have to be very comfortable with the knowledge to present it in a way that can be learnt from. [Year 2 student]*

*It is true that teaching others is the best way to truly understand a topic and VI (vertical integration) gave me ample opportunity to do this. [Year 4 student]*

#### Leadership and teamwork

Senior students (years 4–6) reflected on the development of their leadership and teamwork skills and their progression from learners to leaders; a transition nurtured by the opportunities for leadership offered by vertical integration. Students also commented on the value of being in teams with colleagues of varying levels of ability and motivation.

*I took on leadership roles in group projects that I may not have done in an evenly experienced team. [Year 4 student]*

*The opportunity to take on various roles within the team–from learning, to leader. [Year 6 student]*

*…vertical learning in Phase 3 (in the clinical setting) was definitely very useful in terms of developing better teamwork. [Year 6 student]*

Some senior leaders independently organised teaching activities, including mock examinations. Mock examinations were seen as integral to examination preparation and highly valued.

*(Vertical integration)…builds a sense of responsibility to teach juniors, which is an ongoing part of professional practice. [Year 6 student]*

*In Phase 3 (years 5 and 6), the years above did a great job preparing the years below for their exams. This included interns helping Year 6 students. [Year 6 student]*

#### Students’ resistance to peer learning

Year 1 students commented on some year 2 students who were not prepared to work collaboratively in vertically integrated groups.

*I found in my group that the second years were arrogant and rarely worked with first years if they could avoid it. This attitude is entirely counterproductive to the aims of vertical integration, as it prevented us from learning alongside the second years and made several first years in our scenario group feel unnecessarily insecure and inadequate. [Year 1 student]*

#### Transmission of inaccurate information

Some junior students were concerned about the accuracy of information and teaching of concepts provided by senior students.

*Medical students are unwilling to reveal their uncertainty on topics they “should” know and propagate misleading information. Similarly in all areas of vertical integration poor information is transmitted from older to younger students. However I feel this is outweighed by the accurate information, which is highly relevant to younger student’s knowledge base. [Year 4 student]*

*Students may provide incorrect or incomplete information. Thus some sessions may be more of a waste of time rather than effective learning. [Year 5 student]*

#### Impediments to learning

A minority of students questionned the pedagogical basis for vertical integration and viewed it as an impediment to their learning.

*The Faculty of Medicine should be ashamed for lumping first and second year students together in an obvious cost-cutting manoeuvre, and attempting to disguise it as ‘Peer-Teaching’ or ‘Near-Peer Teaching.’ [Year 4 student]*

## Discussion

This evaluation of one of the most unique aspects of the UNSW Medicine program, the explicit vertical integration of learners, has demonstrated three important results that are relevant not only for medical education, but also for other professional higher education programs. First, an instructional design that purposely mixes students one year apart within the same courses has led to remarkably positive effects for most students’ experience of learning. The overwhelming majority of students found the experience satisfactory, and students at both levels identified significant benefits for their own learning. Secondly, the design facilitates development of leadership qualities within a team, by providing defined experiences of being both year 1 and year 2 team members. Thirdly, structured vertical integration of learners has engendered a deep culture of near-peer teaching in the student cohort, a culture that has led to widespread student-initiated across year teaching.

Although the literature includes examples of evaluations of peer learning in various educational contexts [9, 17, 23] our experience of a *program wide* mixing of students from adjacent cohorts into single courses that involve the same learning and teaching activities and the same assessments is unique. An early evaluation of our students’ experience of learner vertical integration within a laboratory class setting was positive [24], and provided reassurance to some skeptical teachers. This much more comprehensive evaluation shows both junior and senior cohorts readily articulate the positivity of this structure, and identify key benefits for their own learning. For junior students, the value of guidance, advice and explanations from near peers is recognized in the literature [19, 25, 26] and this is apparent in our medical students. Comments and responses by senior members of learning teams underline the axiom attributed to Joubert, that ‘to teach is to learn twice’. In preparing to teach, the teacher enters into a process where they review, reorganize and simplify their own knowledge in order to scaffold learning for their peers, and it is through this process that their learning is deepened [17, 27, 28].

The experience of peer learning was not positive for all students. Some students felt senior students were not interested in helping or teaching them, whilst others indicated their learning slowed to accommodate students in junior years. With over 1600 students in the UNSW Medicine program drawn from a variety of secondary learning environments, it is possible that some students felt that peer learning did not suit their learning style; some students may have had previous positive experiences of peer learning, whilst others may have had no experience or negative past experiences. Moreover, medical students are a competitive group and some may have felt that peer learning did not support their full learning potential. In future, it will be important to communicate the educational rationale that underpins peer learning to students, and the importance peer learning will have for their medical careers, where they will be working in teams, which will be vertically integrated with senior and junior members.

An important issue we explored was whether there were any negative effects on academic performance by deliberate mixing of near-year cohorts. As shown in Table 4, although year 1 students had higher failure rates than year 2 students in the vertically-integrated HM course, these rates were not different than the respective rates for year 1 and year 2 students in the non-vertically integrated BGD courses. Taken together, the results shown in Tables 4 and 5 suggest that year 2 students outperform year 1 students when examined in vertically-integrated courses, but this is more likely to result from the year 2 students’ prior learning and advanced academic skills than whether the course is vertically-integrated or non-vertically integrated. We conclude that the beneficial effects on learning for both junior and senior cohorts articulated in student responses comes at no significant detriment to examination performance.

Previous evaluations of the UNSW Medicine program have shown that rather than being a personal attribute, the capability of teamwork can be readily learned and assessed with appropriate curriculum and assessment design [3, 5, 7, 8, 29]. Indeed, new graduates of the UNSW Medicine program have better developed teamwork capabilities than any other previously evaluated junior doctor [29]. This report shows a new aspect of teamwork development, that of leadership within a team context that has led to a remarkable expansion of student-initiated near-peer teaching. In her systematic review of peer teaching and learning in health professional students within clinical placements, Secomb [23] found some evidence that peer learning led to the development of leadership skills, as the role of the near-peer teacher is not only to present knowledge, but also to organise, communicate and facilitate within a group of peers. These skills are equally applicable when communicating with and educating patients [9, 30, 31].

A limitation of this study is the relatively small sample size that may be a result of using an online questionnaire asking students to comment retrospectively on their learning experiences. The small sample size may also reflect students’ reluctance to be involved in evaluation studies. As the questionnaire is anonymous, we are unaware of the characteristics of the non-responders and it is unclear to what extent these results can be extrapolated to the wider medical student population, but in previous evaluations which are not anonymous the responders and non-responders are generally closely matched.

## Conclusions

Teaching within Medicine programs is designed to graduate doctors who are ready and competent for clinical practice. For example, clinical skills and knowledge are taught and assessed for competency whereas work place practices have generally been passed onto students as part of a culture. By teaching in a vertically integrated structure the culture of working in teams is explicitly modelled and fostered for students in preparation for their working career. Moreover, senior students seem to feel a professional ‘obligation’ to teach their junior colleagues in much the same way that practicing clinicians feel a responsibility to teach medical students, much of which is done for no financial gain. It is important that medical students see teaching as an integral and rewarding component of their role as clinicians and that teaching skills are incorporated early into their undergraduate medical curriculum [26, 30, 32].

This evaluation demonstrates the many positive features of peer learning that have encouraged collaborative learning and the development of teamwork capabilities, in particular leadership qualities within teams. A remarkable benefit has been the development of widespread student-initiated near-peer teaching between senior student teachers and more junior student learners. We believe our approach provides a unique model that could be applied to other health professional educational programs.
